# Outcome of non-surgical dietary treatment with or without lactulose in dogs with congenital portosystemic shunts

**DOI:** 10.1080/01652176.2020.1745928

**Published:** 2020-04-03

**Authors:** Robert P. Favier, Eline de Graaf, Ronald J. Corbee, Anne Kummeling

**Affiliations:** Department of Clinical Sciences of Companion Animals, Faculty of Veterinary Medicine, Utrecht University, Utrecht, the Netherlands

**Keywords:** canine, congenital portosystemic shunt, non-surgical treatment, survival time, quality of life

## Abstract

**Background:**

Congenital portosystemic shunts (CPSS) are vascular anomalies, allowing portal blood to bypass the hepatic parenchyma, thereby accumulating toxic substances such as ammonia in the systemic circulation resulting in hepatic encephalopathy.

**Aim:**

To evaluate the outcome of non-surgically treated dogs with a CPSS.

**Methods:**

Case records of 78 dogs with a single congenital CPSS confirmed by ultrasound and/or computed tomography between September 2003 and February 2015 were reviewed. Median age at diagnosis of CPSS in dogs was 10.8 months (range 2–133 months). Non-surgical treatment was started as an adjusted diet (a diet restricted in protein) with or without lactulose. Owners were contacted by telephone to determine survival time and presumed cause of death, if applicable. In addition, a questionnaire was used to retrospectively assess quality of life (QoL) and CPSS scores in 37 dogs before and during non-surgical treatment. Differences between Kaplan–Meier curves were tested by a Log rank test.

**Results:**

Overall estimated median survival time (EMST) was 38.5 months (range 1 day − 91 months; 78 dogs). No significant differences between EMSTs were found between dogs with extra- (*n* = 48) or intrahepatic (*n* = 29) shunts, nor between treatment with only an adjusted diet, or an adjusted diet combined with lactulose. During non-surgical treatment, significant improvement in perceived QoL and CPSS scores were found (*P* < 0.01).

**Conclusion:**

Our study demonstrated that an overall median EMST of 3.2 years was reached and that owners retrospectively perceived that non-surgical treatment resulted in an improved QoL and clinical performance, irrespective of intrahepatic or extrahepatic CPSS location.

## 1. Introduction

Congenital portosystemic shunts (CPSS) are vascular anomalies, allowing portal blood to bypass the hepatic parenchyma and directly enter the systemic circulation (Mankin 2015). Extrahepatic CPSS (EHPSS) and intrahepatic CPSS (IHPSS) are likely to have a different genetic profile (Van den Bossche and van Steenbeek 2016). EHPSS predominantly affects small breed dogs, whereas in large-breed dogs IHPSS are more common (Hunt 2004). Toxic substances such as ammonia, accumulate in the systemic circulation and are responsible for the development of hepatic encephalopathy (HE) in dogs with CPSS (Butterworth et al. 1987; Perazzo et al. 2012), potentiated by an increased blood manganese concentration and a systemic inflammatory response syndrome (SIRS) (Gow et al. 2010, 2012; Tivers et al. 2014; Gow 2017). Besides HE, vomiting, diarrhea, and ammonium-urate urolithiasis are frequently observed (Broome et al. 2004; Berent and Tobias 2009). These clinical signs of portosystemic shunting often affect quality of life (QoL) and survival. Surgical intervention is frequently recommended for animals with CPSS because of improved survival and QoL compared with non-surgical treatment (Greenhalgh et al. 2010; Tivers et al. 2017). Different methods for surgical shunt occlusion have been investigated (Berent and Tobias 2009; Tivers et al. 2012; Greenhalgh et al. 2014; Mankin 2015; Tivers et al. 2017). Unfortunately, surgery is not always possible for anatomical or financial reasons, or concerns regarding acute mortality. Non-surgical treatment, unlike surgery, does not affect the portal blood flow into the systemic circulation (Watson and Herrtage 1998; Mankin 2015), but aims to control clinical signs by reducing the systemic plasma concentration of toxic substances (Watson and Herrtage 1998; Proot et al. 2009). The latter can be achieved by feeding a diet that has a lower amount of protein (approximately 40 g per 1,000 kcal metabolizable energy) with a high branched-chain amino acids to aromatic amino acids ratio (Proot et al. 2009). Furthermore, sources of soluble fiber, in the diet or as supplement (e.g. lactulose), reduce ammonia uptake from the gastrointestinal tract by an acidifying effect that shifts ammonia into ammonium which is not absorbed, and by a laxative effect that ensures rapid expulsion of bacteria and ammonia (Van Leeuwen et al. 1988; Bajaj 2008). Antimicrobial treatment might exert similar effects (Watson and Herrtage 1998; Berent and Tobias 2009; Greenhalgh et al. 2014; Mankin 2015). Only a few studies have described the long-term follow-up of non-surgical treatment of CPSS patients comprising relatively small groups of dogs (Watson and Herrtage 1998; Faverzani et al. 2003; Tivers et al. 2012; Greenhalgh et al. 2014; Tivers et al. 2017). For assessment of clinical performance in dogs with CPSS, a CPSS score was used that was published by Bristow et al. and includes frequency and severity of clinical signs (Bristow et al. 2019). The aim of this study was to report outcome, described as survival time, clinical performance (CPSS score), and QoL in a cohort of dogs with a CPSS that was not treated surgically.

## 2. Materials and methods

### 2.1. Animals

Case records of dogs with a single congenital CPSS referred to the Department of Clinical Sciences of Companion Animals (DCSCA), Utrecht University, the Netherlands, between September 2003 and February 2015 were reviewed. Cases were selected in which the CPSS was confirmed by ultrasound and/or computed tomography, and there was no surgical intervention, or there was surgical exploration of the shunt but no attenuation, and the patient received some form of non-surgical treatment. Non-surgical treatment was started as an adjusted diet (a diet restricted in protein) with or without lactulose, for at least one month or until death if the dog’s survival time was less than one month. Dogs that received an adjusted diet for longer than one month were assigned to one of two treatment groups: ‘adjusted diet’ and ‘adjusted diet in combination with lactulose’. Animals were assigned to the ‘adjusted diet’ group if they had received a diet restricted in protein, which was a commercial hepatic diet, a commercial renal diet, or a home cooked diet restricted in protein. Dogs were assigned to the ‘adjusted diet in combination with lactulose’ group if they had received lactulose in combination with an adjusted diet. If treatment had changed over time, the patient was assigned to the group of treatment that they received last. As antibiotics were not routinely prescribed, their usage was not included as treatment. Patient description, medical history, age at diagnosis, and intrahepatic or extrahepatic shunt location, were obtained from the medical records.

### 2.2. Follow-up

Owners were contacted by telephone to determine survival time and presumed cause of death, if applicable. Referring veterinarians were contacted if the owners did not respond. Owners were asked to confirm that their pet had received non-surgical treatment, and what the nature of the last treatment consisted of. In deceased dogs, the cause of death was recorded to establish if death was suspected to be related to CPSS or not. If non-surgical treatment for at least one month had been confirmed, permission was requested to send a questionnaire for scoring the occurrence and frequency of clinical signs (scored on an ordinal scale from 1, ‘never’, up to 5, ‘daily’) and QoL (also scored on an ordinal scale from 1, ‘worst imaginable’, up to 5, ‘best imaginable’) before and during non-surgical treatment (Supplementary file no. 1). The owners were asked to indicate an average score of clinical signs and QoL over the follow-up period that the dog received last treatment. CPSS scores were calculated based on assessment of 17 clinical signs according to Bristow et al. (2019), resulting in a score between 0 and 110.

### 2.3. Statistical analysis

Statistical analysis was performed using IBM SPSS Statistics version 22 and 25 (IBM Corporation). Survival analysis was based on a Kaplan–Meier curve. Survival time was defined as the time between confirmed diagnosis and death. Patients that died of CPSS-related causes were counted as events. If the cause of death was considered to be unrelated, patients were censored (withdrawn from further survival analysis). Differences between Kaplan–Meier curves were tested by a Log rank test. Survival data are presented as estimated median survival times (EMST) [95% confidence interval (CI)]. After confirming that data were not normally distributed, Wilcoxon signed rank tests for paired data were performed to compare QoL perception by owners and CPSS scores before treatment and at follow-up. A *P*-value <0.05 was considered significant.

## 3. Results

### 3.1. Animals

Seventy-eight dogs with confirmed CPSS met the inclusion criteria. Breed and shunt location (EHPSS, IHPSS) are presented in Table 1. In seven dogs an initial attempt was made to surgically ligate the shunt, but no ligation or attenuation was possible because of severe hypoplasia or aplasia of the portal vein cranial to the shunt (5 dogs with EHPSS), hepatic parenchymal bleeding (1 IHPSS dog) or recurrent severe systemic arterial hypotension during dissection of the shunt (1 IHPSS dog). Non-surgical treatment was already started prior to surgery and was continued postoperatively, and therefore these dogs were included. Reasons for not intervening surgically were financial restrictions, relatively old age at time of diagnosis, lower odds for successful surgical treatment of IHPSS (Tivers et al. 2017; Berent and Tobias 2018), and improvement during non-surgical treatment.

**Table 1.** Shunt location (EHPSS, IHPSS) and breed distribution of 78* dogs with CPSS receiving non-surgical treatment only.

Breeds with EHPSS	*N*	Breeds with IHPSS	*N*
Yorkshire terrier	6	Bernese mountain dog	4
Cairn terrier	5	Nova Scotia duck tolling retriever	4
Chihuahua	5	Mixed breed	2
Miniature schnauzer	5	Dobermann	2
Jack Russell terrier	4	Golden retriever	2
Shih Tzu	4	Labrador retriever	2
Maltese	3	Borzoi	1
Mixed breed	2	Basset hound	1
Dachshund	2	Belgian shepherd dog	1
Labrador retriever	2	Cavalier King Charles spaniel	1
Cavalier King Charles spaniel	1	Chihuahua	1
English springer spaniel	1	English cocker spaniel	1
Golden retriever	1	French bulldog	1
Keeshond large	1	Grand Basset Griffon Vendéen	1
Lhasa Apso	1	Jack Russell terrier	1
Miniature poodle	1	Maltese	1
Norfolk terrier	1	Rhodesian ridgeback	1
Pug	1	Scottish terrier	1
Schapendoes	1	Weimaraner	1
West Highland white terrier	1		
Total number	48		29

*In one dog (Cairn terrier), a CPSS was diagnosed but the extra- or intrahepatic location of the shunt could not be established.

Forty-eight dogs were diagnosed with EHPSS and 29 dogs with IHPSS. In one dog, only CPSS was diagnosed but the extra- or intrahepatic location of the shunt could not be established. More EHPSS were seen in the small breed dogs (<10 kg; 42/48) compared to large breed dogs (>10 kg; 6/48). In contrast, 22 large breed dogs were diagnosed with IHPSS, whereas IHPSS was diagnosed in 7 small breed dogs. Median age at diagnosis of CPSS in dogs was 10.8 months, (range 2–133 months).

### 3.2. Clinical signs

The majority of dogs (61/78) showed clinical signs of dysfunction of multiple organ systems. Clinical signs at the time of diagnosis were related to the central nervous system, gastrointestinal tract (i.e. vomiting and diarrhea), and lower urinary tract (in some cases with urethral obstruction caused by urolithiasis, in most cases confirmed as ammonium urates). Other clinical signs at the time of diagnosis were retarded growth, polyuria/polydipsia (pu/pd), and pale mucous membranes (Table 2). Two dogs did not show clinical signs according to the owners. One of them was a 2-month-old puppy, which was diagnosed with a CPSS during a routine screening, while the other dog was diagnosed with a CPSS after a pre-anesthetic blood examination showing increased plasma liver biochemical analyte values.

**Table 2.** Clinical signs before and/or at time of diagnosis in 78 dogs with CPSS receiving non-surgical treatment only.

Clinical signs^a^	*N*	Percentage^b^
Neurological signs (head pressing, circling, ataxia, apparent blindness, sopor and disorientation)	52	67%
pu/pd	37	47%
Gastrointestinal signs (vomiting, diarrhea, anorexia and low body condition score)	25	32%
Urinary tract disease with confirmed ammonium urates	17 11	22% 14%
Retarded growth	13	17%
Pale mucous membranes	3	4%
No clinical signs present	2	3%

^a^Dogs can show more than one clinical sign.

^b^Percentages were calculated by number (*N*)/78 * 100%.

### 3.3. Non-surgical treatment

**Figure 1.**
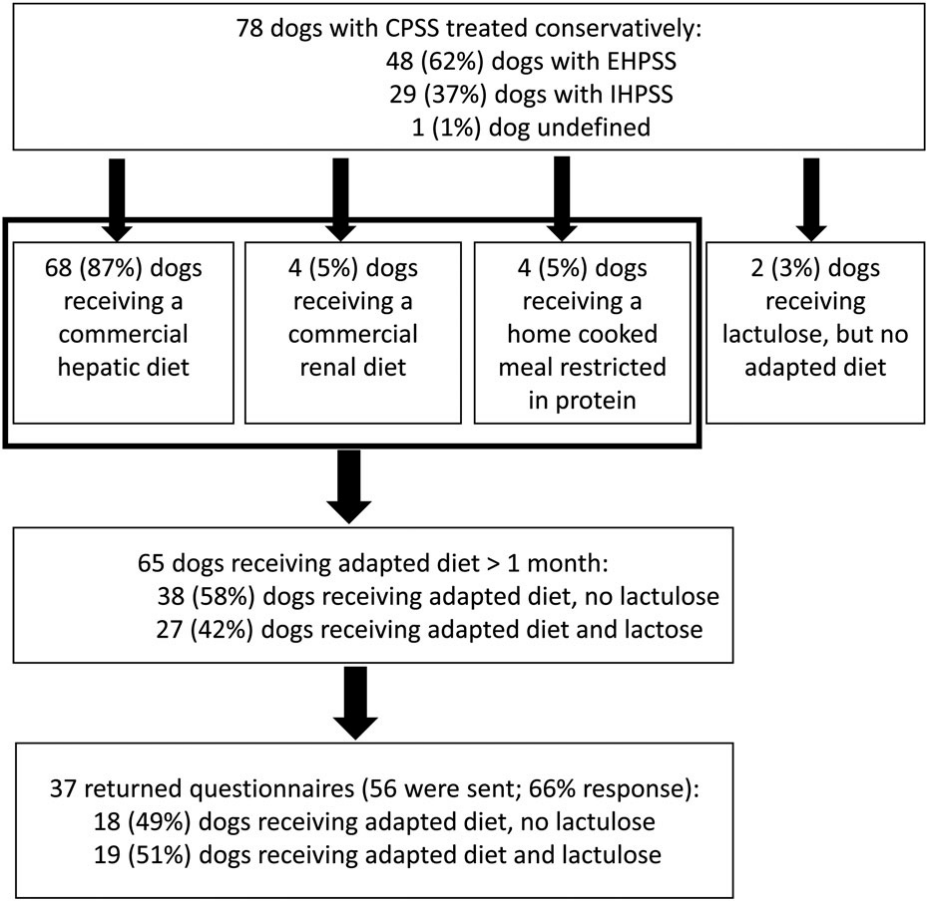
Follow-up of 78 dogs with CPSS (2003–2015) treated non-surgically. CPSS, congenital portosystemic shunts; EHPSS, extrahepatic congenital portosystemic shunt; IHPSS, intrahepatic congenital portosystemic shunt.

Sixty-eight dogs received a commercial hepatic diet, four dogs a commercial renal diet, four dogs a home cooked diet restricted in protein, and in two dogs lactulose was started, but the diet was not adapted after diagnosing the CPSS (Figure 1). In 11 dogs, it could not be established if they received only an adjusted diet or an adjusted diet combined with lactulose. For 65 dogs fed an adjusted diet it was confirmed they had received this diet for over a month, and 27 of them were treated with lactulose as well during this period.

### 3.4. Survival time

**Figure 2.**
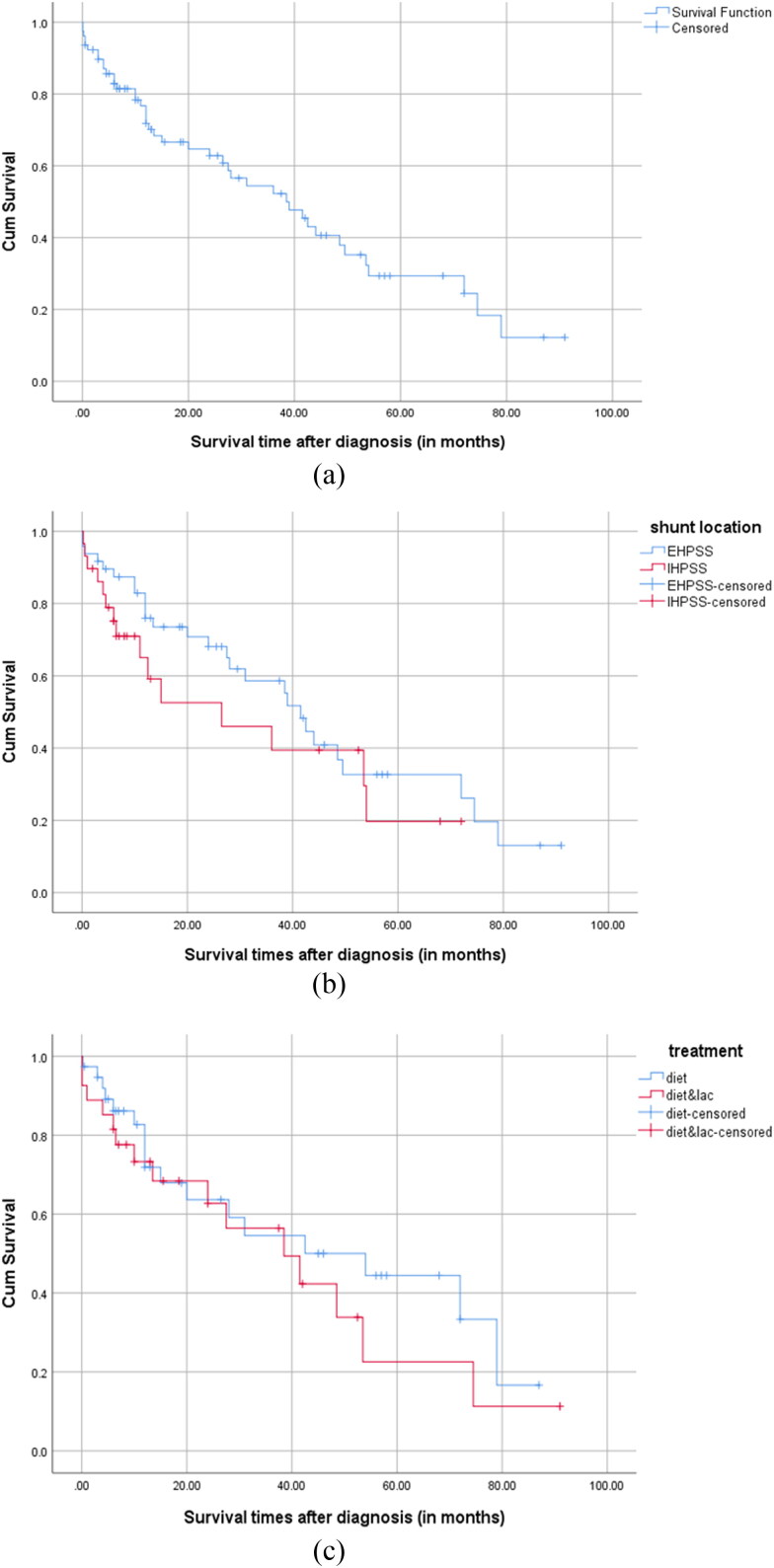
Kaplan–Meier curves for the survival times of (A) all non-surgically treated dogs, *n* = 78 dogs (overall EMST 38.5 months; 95% CI 24.452.6), (B) non-surgically treated dogs per shunt location (*n* = 77) [EHPSS (EMST 41.5 months; 95% CI 35.2–47.8) vs. IHPSS (EMST 26.5 months; 95% CI 0–53.6), not significant (*P* = 0.12)], and (C) dogs non-surgically treated for >1 month per treatment group *n* = 65 [adjusted diet (*n* = 38) (EMST 54.0 months; 95% CI 18.2–89.8) vs. adjusted diet with lactulose (*n* = 27) (EMST 38.5 months; 95% CI 15.9–61.1), not significant (*P* = 0.08)]. Censored cases were withdrawn from further survival analysis because of death unrelated to portosystemic shunting or ending the follow-up period.

Survival times after diagnosis were estimated for the entire group (*n* = 78), for intrahepatic and extrahepatic shunts (*n* = 77), and in both treatment groups of dogs receiving an adjusted diet for more than 1 month (*n* = 65) (Figure 2A–C). Portosystemic shunting related mortality was 42 of 78 dogs (54%), and an additional five dogs died due to unrelated causes. Most dogs were euthanized (*n* = 34), whereas 13 dogs died spontaneously. The survival times ranged from 1 day up to 6.6 years (median 13 months). The overall EMST after diagnosis for all dogs was 38.5 months or 3.2 years (range 0–91 months; 95% CI 24.4–52.6). Neither shunt location [EHPSS EMST 41.5 months (95% CI 35.2–47.8), IHPSS EMST 26.5 months (95% CI 0–53.6), *P* = 0.28], nor treatment group [only adjusted diet EMST 54.0 months (95% CI 18.2.5–89.8), adjusted diet and lactulose EMST 38.5 months (95% CI 15.9–61.1), *P* = 0.39] differed significantly in EMST. Median follow-up time of survivors (*n* = 31) was almost 4 years (47 months, range 3–91 months). The median age at shunt-related death was 6.3 years (range 2 months–12.5 years; *n* = 42).

### 3.5. Clinical performance and QoL

Questionnaires were sent out to 56 dog owners, of which 37 returned the questionnaire. The EHPSS/IHPSS proportion was 20/17, from which 10 and 8 dogs respectively, were deceased at time of follow-up. From the 37 returned questionnaires, 18 dogs (11 dogs with EHPSS, 7 dogs with IHPSS) received an adjusted diet and 19 dogs (9 dogs with EHPSS, 10 dogs with IHPSS) received an adjusted diet and lactulose (Figure 1). CPSS scores for the whole group (*n* = 37) were significantly lower during (median 18, range 0–70) than before treatment (median 35, range 4–72) (*P* = 0.01). Overall QoL scored by the owners significantly improved at the time of follow-up (median 2, range 1–5 before treatment versus median 4, range 1–5 during treatment, *n* = 37; *P* < 0.001).

## 4. Discussion

This retrospective study aimed to determine outcome for dogs with CPSS that received non-surgical treatment (a diet restricted in protein and/or lactulose) only. This study found an overall EMST of 3.2 years (range 0–91 months) in a group of 78 dogs with CPSS treated only non-surgically, with no significant difference in EMST between dogs with EHPSS and IHPSS. In the vast majority of cases, dogs died or were euthanized because of clinical signs of HE that were directly related to their CPSS.

Only two other studies reported survival times for dogs with CPSS treated non-surgically only. Watson and Herrtage (1998) described the follow-up in 23 dogs, of which 14 had been euthanized with a median survival time of 6 months. Nine dogs were still alive at the end of that study resulting in a median period of 51 months (range 36–96 months) [4.3 years (range 3–8 years)] between diagnosis and end of the study (Watson and Herrtage 1998). Greenhalgh et al. (2014) found an EMST of 27.4 months (2.3 years) for 24 dogs that died or had been euthanized during the study period and the three remaining dogs were lost at 5.5–96.7 months (0.5–8.1 years) of follow-up. These authors did not find a significant difference of EMST between EHPSS and IHPSS either. Both studies (Watson and Herrtage 1998; Greenhalgh et al. 2014) had a smaller study population and no overall EMST was reported. The median survival time of the deceased dogs reported here (13 months) is within the range of survival times in the two studies.

Greenhalgh et al. (2014) compared the survival rate between dogs with CPSS treated non-surgically versus dogs treated surgically and found that the survival rate was significantly improved in surgically treated dogs (hazard ratio 8.11; 95% CI 4.20–15.66). Although an overall EMST of 38.5 months (more than 3 years) for non-surgically treated dogs appears to be encouraging, its value for an individual dog depends on the age at diagnosis and the age of diagnosis shows a wide range in this study. At an age at diagnosis of older than 5 years, a life expectancy of another 3.5 years may be acceptable to the owner, whereas for young dogs at time of diagnosis, a survival time of 3.5 years can be disheartening.

Besides survival time, it is also relevant to estimate how old dogs with a CPSS may become with non-surgical treatment. Although the age that these dogs achieve may also depend on shunt anatomy, age at initial signs and age at diagnosis (Watson and Herrtage 1998; Berent and Tobias 2018), this study found a median age at death of 6.3 years with non-surgical treatment, indicating a reasonable life span. Surgical attenuation of the shunt probably still is the preferred treatment to achieve a longer life span (Greenhalgh et al. 2014).

To the authors’ knowledge this is the first study in which a comparison for survival has been made between diet and diet in combination with lactulose as non-surgical treatment. During the study period of 11 years, different preferences in additional treatment with lactulose were seen. Dogs additionally treated with lactulose probably received diverse dosages, but only lactulose usage was considered for assignment to the adjusted diet plus lactulose treatment group. As the usage of lactulose did not result in a significant longer EMST, it might be concluded that lactulose does not seem to be necessary in a dog that is doing clinically well on only an adjusted diet. It can be used, however, temporarily to stabilize a dog with severe signs of HE, or when an adjusted diet alone proves to be insufficient. Although no difference was found, it might have been possible that lactulose was prescribed only to dogs with more severe clinical signs, which might have extended these patients’ survival time equivalent to that of milder cases requiring only dietary management. Due to small sample size, variable usage and dosages of lactulose, the results should be interpreted with caution.

Besides survival, outcome after treatment is also determined by QoL and clinical performance. Bristow et al. (2019) promoted the use of a consistent outcome assessment tool in dogs with CPSS and designed a questionnaire to compare clinical outcome of treatment in the form of a direct QoL score (using a 10-cm visual analog scale, which was converted to a score from 0 to 100) and a CPSS score (depending on frequency of widely recognized clinical signs of portosystemic shunting, resulting in a score from 0 to 110). According to the study of Bristow et al., both QoL and CPSS scores were necessary to assess owner-assessed outcome. After surgical ligation of CPSS, CPSS scores decreased but remained relatively high compared to healthy controls. However, QoL scores improved in surgically treated dogs to similar levels as in healthy dogs (Bristow et al. 2019). In the current study, median CPSS score before treatment (35) was comparable to the pretreatment scores in the study of Bristow et al. (34 in intrahepatic and 39 in extrahepatic CPSS). Although the overall median CPSS score (18) was significantly lower during treatment than before (35), scores appear to remain higher after non-surgical treatment than after surgical ligation, as Bristow et al. reported median postoperative scores of 3 in EHPSS and 9 in IHPSS. Also, QoL scored relatively low after non-surgical treatment (68 after converting to a 0–100 scale), compared to 94 and 96 in the study of Bristow et al.

The composition of our study group might be biased due to several causes. Firstly, if animals were older at time of diagnosis, owners might have been more reluctant to choose surgical treatment. This could have led to an overrepresentation of older dogs. Furthermore, the location or type of shunt or diameter may be responsible for a later onset of clinical signs (e.g. porto-azygos shunts), leading to a different distribution of shunt type in dogs treated non-surgically.

Secondly, IHPSS (especially the right sided and central divisional shunts) are more difficult and more costly to attenuate surgically (Tivers et al. 2017; Berent and Tobias 2018), resulting in an overrepresentation of intrahepatic shunts in the non-surgically treated dogs. In our study, the IHPSS incidence of 37% was in line with other studies, in which an IHPSS incidence of 13–33% has been found (Berent and Tobias 2009, 2018). During the same period, the IHPSS incidence in dogs that underwent surgical attenuation in our clinic (170 dogs) was 19%, which indeed appears to be lower than 37%.

Thirdly, because of possible long intervals between contacts for some owners, recall bias might have been present, leading to more unreliable results. Median recall time (time elapsed between starting medical treatment and completing the questionnaire) was 12.5 months for all dogs and 4.3 years for deceased dogs. Eleven of 78 owners stated they did not want to participate in the questionnaire as they were elderly people and suggested that they would not remember certain details as well as desired. Despite this possible bias, the same method has been used in other studies (Watson and Herrtage 1998; Greenhalgh et al. 2014; Bristow et al. 2019).

Lastly, owners are most often the ones to request euthanasia when they consider the QoL of their pet to be insufficient, so making assessment of the QoL is important. With their assessment and decision for euthanasia, the owners directly influence the survival time, as most dogs in our study were euthanized before dying from the disease. Besides, as the disease has a fluctuating course with intermittent clinical signs, it is even more difficult and variable to determine clinical performance, QoL, and to decide when to euthanize a dog. By scoring clinical performance and QoL not at one moment in time, but what was perceived by the owner during the entire period that the dog received the assigned medical treatment, an attempt was made to mediate this fluctuation.

In conclusion, when surgical treatment is not an option, non-surgical treatment may be an opportunity to improve QoL and clinical performance in most dogs with CPSS. Definitive conclusions regarding the effect of treatment or shunt location (EHPSS, IHPSS) on EMST and clinical outcome require prospective studies with larger populations of dogs and randomized treatment groups.

## Supplementary Material

Supplemental Material
