# Supplementary material for: Outcome of non-surgical dietary treatment with or without lactulose in dogs with congenital portosystemic shunts
Source: Vet Q. 2020 Apr 3;40(1):108–14. doi: 10.1080/01652176.2020.1745928 (PMC7170303; doi:10.1080/01652176.2020.1745928)
Supplement: Supplemental Material [file TVEQ_A_1745928_SM6689.docx]

**Questionnaire to assess outcome in non-surgically treated dogs with a congenital portosystemic shunt**

Part 1 before treatment: without diet / without medication

**1: How often approximately did your dog show the following signs?**

**(before any treatment was started by a veterinarian)**

Please mark the correct answer with a color or underlining.

**a: Seizures** (*or epilepsy*)

Never Less than once a month Monthly Weekly Daily

How unpleasant was this for your pet?

with 1 representing not unpleasant at all and 5 severely unpleasant

1 2 3 4 5

**b: Head pressing** *(pressing its head against a wall, or standing very close to a wall without moving)*

Never Less than once a month Monthly Weekly Daily

How unpleasant was this for your pet?

1 2 3 4 5

**c: Circling** *(walking around in circles)*

Never Less than once a month Monthly Weekly Daily

How unpleasant was this for your pet?

1 2 3 4 5

**d: Disorientation** *(looks like it has lost its way, does not know what to do or where to go)*

Never Less than once a month Monthly Weekly Daily

How unpleasant was this for your pet?

1 2 3 4 5

**e: Aggression** *(biting, snapping or growling without any reason)*

Never Less than once a month Monthly Weekly Daily

How unpleasant was this for your pet?

1 2 3 4 5

**f: Collapse** *(fainting, to lose consciousness or drop down)*

Never Less than once a month Monthly Weekly Daily

How unpleasant was this for your pet?

1 2 3 4 5

**g: Wobbliness** *(having difficulty to walk normally, walk like a drunken man, falling over)*

Never Less than once a month Monthly Weekly Daily

How unpleasant was this for your pet?

1 2 3 4 5

**h: Unresponsive/ vacant episodes** *(less or not reacting to the environment, drowsing)*

Never Less than once a month Monthly Weekly Daily

How unpleasant was this for your pet?

1 2 3 4 5

**i: (Apparent) blindness** *(walking against obstacles or walls)*

Never Less than once a month Monthly Weekly Daily

How unpleasant was this for your pet?

1 2 3 4 5

**j: Lethargy/ weakness** *(lies down quickly, does not have enough energy, sleeps a lot)*

Never Less than once a month Monthly Weekly Daily

How unpleasant was this for your pet?

1 2 3 4 5

**k: Vomiting** *(throwing up food, bile or gastric juice)*

Never Less than once a month Monthly Weekly Daily

How unpleasant was this for your pet?

1 2 3 4 5

**l: Diarrhoea** *(thinner stool)*

Never Less than once a month Monthly Weekly Daily

How unpleasant was this for your pet?

1 2 3 4 5

**m: Inappetance** *(No or less appetite)*

Never Less than once a month Monthly Weekly Daily

How unpleasant was this for your pet?

1 2 3 4 5

**n: Difficulty urinating** *(straining or pressing during urination, very frequent urination)*

Never Less than once a month Monthly Weekly Daily

How unpleasant was this for your pet?

1 2 3 4 5

**o: Blood in urine** *(red urine)*

Never Less than once a month Monthly Weekly Daily

How unpleasant was this for your pet?

1 2 3 4 5

**p: Excessive saliva drooling** *(spontaneous salivating)*

Never Less than once a month Monthly Weekly Daily

How unpleasant was this for your pet?

1 2 3 4 5

**q: Excessive drinking** *(in combination with more frequent and excessive urination)*

Never Less than once a month Monthly Weekly Daily

How unpleasant was this for your pet?

1 2 3 4 5

**2: Did your pet suffer from bladder stones or urinary obstruction that required you to take him/her to your vet for diagnosis/treatment?**

If so, please give details below?

Yes No

**3: Did you consider your dog to be small / underweight for their breed and age before treatment?**

Yes No Not sure

**4:** **How active was your dog?**

(with 1 representing not active at all and 5 maximal active)

1 2 3 4 5

**5: How willing was your pet to:**

(with 1 representing not willing at all and 5 could not be more willing)

**a: play?**

1 2 3 4 5

**b: interact (contact) with you as the owner?**

1 2 3 4 5

**c: exercise?**

1 2 3 4 5

**d: interact (contact) with other dogs?**

1 2 3 4 5

**6. Did your dog have a surgery in which the shunt was narrowed or closed?**

No Yes

**If not, what was/were the reason(s) for not choosing surgery?**

0 young age (not 6 months yet)

0 old age (older than 5 years when diagnosed)

0 risk of surgery

0 the surgeon advised not to operate

0 costs

0 clinical improvement with medical treatment and/or diet

0 otherwise:

*If otherwise, could you please specify below:*

**7a: Did your pet get treated with medication (for example lactulose) and/or a special diet for liver disease?**

**If so, what was used?**

(Examples of diets for supporting liver function are ‘hepatic’ from Royal Canin and ‘l/d’ from Hills.)

No Diet Lactulose Diet and lactulose

Other medication /diet

*please specify below:*

**7b. Have any changes occurred in /during this treatment?** (For example, a change in diet, quitting lactulose or adding lactulose)

No Yes

*If yes, could you specify what these changes were and when they occurred?*

Part 2 during treatment: with diet and/or medication

**1: If your pet was/is given a special diet, could you indicate if and how much improvement was or is seen?**

(with 1 representing no improvement at all and 5 maximal improvement)

1 2 3 4 5

**2: If your pet was given a special diet and/or medication, do you think this had an ADVERSE effect on its quality of life?**

(with 1 representing no adverse effect at all and 5 maximal adverse effect)

1 2 3 4 5

**3: How often showed your pet the following symptoms?** (during treatment)

**a: Seizures** (*or epilepsy*)

Never Less than once a month Monthly Weekly Daily

How unpleasant is/was this for your pet?

with 1 representing not unpleasant at all and 5 severely unpleasant

1 2 3 4 5

**b: Head pressing** *(pressing its head against a wall, or standing very close to a wall without moving)*

Never Less than once a month Monthly Weekly Daily

How unpleasant is/was this for your pet?

1 2 3 4 5

**c: Circling** *(walking around in circles)*

Never Less than once a month Monthly Weekly Daily

How unpleasant is/was this for your pet?

1 2 3 4 5

**d: Disorientation** *(looks like it has lost its way, does not know what to do or where to go)*

Never Less than once a month Monthly Weekly Daily

How unpleasant is/was this for your pet?

1 2 3 4 5

**e: Aggression** *(biting, snapping or growling without any reason)*

Never Less than once a month Monthly Weekly Daily

How unpleasant was this for your pet?

1 2 3 4 5

**f: Collapse** *(fainting, to lose consciousness or drop down)*

Never Less than once a month Monthly Weekly Daily

How unpleasant is/was this for your pet?

1 2 3 4 5

**g: Wobbliness** *(having difficulty to walk normally, walk like a drunken man, falling over)*

Never Less than once a month Monthly Weekly Daily

How unpleasant is/was this for your pet?

1 2 3 4 5

**h: Unresponsive/ vacant episodes** *(less or not reacting to the environment, drowsing)*

Never Less than once a month Monthly Weekly Daily

How unpleasant is/was this for your pet?

1 2 3 4 5

**i: (Apparent) blindness** *(walking against obstacles or walls)*

Never Less than once a month Monthly Weekly Daily

How unpleasant is/was this for your pet?

1 2 3 4 5

**j: Lethargy/ weakness** *(lies down quickly, does not have enough energy, sleeps a lot)*

Never Less than once a month Monthly Weekly Daily

How unpleasant is/was this for your pet?

1 2 3 4 5

**k: Vomiting** *(throwing up food, bile or gastric juice)*

Never Less than once a month Monthly Weekly Daily

How unpleasant is/was this for your pet?

1 2 3 4 5

**l: Diarrhea** *(thinner stool)*

Never Less than once a month Monthly Weekly Daily

How unpleasant is/was this for your pet?

1 2 3 4 5

**m: Inappetance** *(no or less appetite)*

Never Less than once a month Monthly Weekly Daily

How unpleasant is/was this for your pet?

1 2 3 4 5

**n: Difficulty urinating** *(straining or pressing during urination, very frequent urination)*

Never Less than once a month Monthly Weekly Daily

How unpleasant is/was this for your pet?

1 2 3 4 5

**o: Blood in urine** *(red urine)*

Never Less than once a month Monthly Weekly Daily

How unpleasant is/was this for your pet?

1 2 3 4 5

**p: Excessive saliva drooling** *(spontaneous salivating)*

Never Less than once a month Monthly Weekly Daily

How unpleasant is/was this for your pet?

1 2 3 4 5

**q: Excessive drinking** *(in combination with more frequent and excessive urinating)*

Never Less than once a month Monthly Weekly Daily

How unpleasant is/was this for your pet?

1 2 3 4 5

**4: Does/did your pet suffer from bladder stones or urinary obstruction that required you to take him/her to your vet for diagnosis/treatment?**

**If so, please give details below?**

Yes No

**5: Would you have considered your dog to be small / underweight for their breed and age during treatment?**

Yes No Not sure

**6:** **How active is/was your dog?**

(with 1 representing not active at all and 5 maximal active)

1 2 3 4 5

**7: How willing was your pet to:**

(with 1 representing not willing at all and 5 could not be more willing)

**a: play?**

1 2 3 4 5

**b: interact (contact) with you as the owner?**

1 2 3 4 5

**c: exercise?**

1 2 3 4 5

**d: interact (contact) with other dogs?**

1 2 3 4 5

**8: Do you feel your dog has improved in body condition during treatment?**

(with 1 representing not improved at all and 5 maximal improvement)

1 2 3 4 5

*How would you estimate the body condition score of your dog before treatment*

*(see attachment)?*

*Score before treatment:*

*How would you estimate the body condition score of your dog during treatment*

*(see attachment)?*

*Score during treatment:*

**9: How much overall improvement was achieved during treatment?**

(with 1 representing no improvement and 5 maximal improvement)

1 2 3 4 5

**10: How satisfied are you with your dog’s response to treatment?**

(with 1 representing not satisfied at all and 5 maximally satisfied)

1 2 3 4 5

**11: How would you score the quality of life of your pet before treatment?**

(with 1 representing the worst imaginable quality of life and 5 the best imaginable quality of life)

1 2 3 4 5

**12: How would you score the quality of life of your pet during treatment?**

(with 1 representing the worst imaginable quality of life and 5 the best imaginable quality of life)

1 2 3 4 5

**13: Do/did you still worry about the health of your pet or your dog’s condition?**

(with 1 representing not worried at all and 5 could not be more worried)

1 2 3 4 5

Thank you very much for your help!
